# Orthorexia nervosa and disordered eating attitudes among Lebanese adults: Assessing psychometric proprieties of the ORTO-R in a population-based sample

**DOI:** 10.1371/journal.pone.0254948

**Published:** 2021-08-26

**Authors:** Souheil Hallit, Anna Brytek-Matera, Sahar Obeid

**Affiliations:** 1 Faculty of Medicine and Medical Sciences, Holy Spirit University of Kaslik (USEK), Jounieh, Lebanon; 2 INSPECT-LB: National Institute of Public Health, Clinical Epidemiology and Toxicology, Beirut, Lebanon; 3 Research and Psychology Departments, Psychiatric Hospital of the Cross, Jal Eddib, Lebanon; 4 Institute of Psychology, University of Wroclaw, Wroclaw, Poland; 5 Faculty of Arts and Sciences, Holy Spirit University of Kaslik (USEK), Jounieh, Lebanon; European University of Rome, ITALY

## Abstract

**Background:**

Previous research demonstrated a relationship between ON and disordered eating symptoms (eating concern, restraint, cognitive preoccupations about body shape and weight) and disordered eating attitudes (DEA). Since screening for orthorexia nervosa is now part of clinical practice, the measurement instruments to be used must be clinically significant, reliable, valid and sensitive to our target population. The main objective of the present study was to confirm the factor structure of the Arabic version of the ORTO-R using a first sample of Lebanese adults and confirm those results on another sample. The secondary objective was to assess sex differences in terms of ON and DEA, as well as to examine whether symptoms of ON were related to DEA in Lebanese adults.

**Methods:**

A total of 783 Lebanese adults was selected to participate in this cross-sectional study (January-May 2018) using a proportionate random sample from all Lebanese governorates.

**Results:**

The mean age of the total sample was 27.78 ± 11.60 years (Min. 18 –Max. 84) (33.5% females) and their mean BMI was 24.36 ± 5.31 kg/m^2^. All items of the ORTO-R were extracted during the factor analysis and yielded a two-factor solution with Eigenvalues > 1 (variance explained  =  50.07%; KMO = 0.570; Bartlett’s sphericity test *p*<0.001; α_Cronbach_ = 0.755). This factor structure was confirmed by a confirmatory factor analysis; the Maximum Likelihood Chi-Square  =  26.894 and Degrees of Freedom  =  8, which gave a χ2/df  =  3.36. The Tucker Lewis Index (TLI) value was 0.914, whereas the standardized root mean square residual (SRMR) value was 0.032. The root mean square error of approximation (RMSEA) value was 0.077 [95% CI 0.046–0.111] (pclose = 0.07) and comparative fit index (CFI) value was 0.967 respectively, indicating a good fit of the model. There was also no measurement invariance between genders. Female gender was significantly associated with lower ORTO-R scores (more orthorexia nervosa) compared to males (B = -0.65; p = 0.026, 95% CI -1.22- -0.08; ɳ^2^ = 0.006). However, no significant difference was found between genders in terms of EAT-26 scores (B = 0.23; p = 0.813, 95% CI -1.66–2.12; ɳ^2^ = 0.0001). Higher ORTO-R scores (lower/ less pathological ON tendencies and behaviors) were significantly related to higher EAT-26 total scores (higher levels of DEA) as well as higher dieting, bulimia and oral control scores in both females (from a weak to a moderate positive correlation) and males (a weak positive correlation).

**Conclusion:**

This cross-sectional population-based study confirmed the factor structure of the Arabic version of the ORTO-R, demonstrated an association between ON and DEA and revealed more ON among females compared to males.

## Introduction

Not yet classified in the Diagnostic and Statistical Manual of Mental Disorders, Fifth Edition (DSM-5) [1] or in the International Classification of Diseases, Eleventh Revision (ICD-11) [2], Orthorexia Nervosa (ON) is defined as obsessive beliefs and compulsive behaviors concerning ’pure’ eating behavior [3]. Those beliefs and behaviors are also accompanied by excessive emotional distress, state of blame and/or concern if the person violates the strict dietary rules, physical and psychosocial impairments in social, professional and/or educational functioning [4–7].

Previous research demonstrated a relationship between ON and disordered eating symptoms (eating concern, restraint, cognitive preoccupations about body shape and weight) and disordered eating attitudes (DEA) [8–13]. DEA are defined as unhealthy behaviors, ranging from firm eating habits aiming to lose/maintain weight, to severe food restriction [14]. A Spanish study demonstrated that the Eating Attitude Test (EAT-26) measures abnormal eating behaviors and four postulated features (social pressure, food preoccupation, purging behaviors and food awareness), with the latter being able to predict orthorexic behaviors [15]. EAT-26 scores were shown to be significantly associated with orthorexia nervosa among university students [16, 17] and the general population [18, 19].

Several socio-demographic factors have been correlated with ON [13, 20]. A systematic review concluded that tendencies towards healthy eating are similar between men and women, however, women have more pathological healthful eating [21]. To note that these results differ according to the scale used to measure ON [21].

Scientific interest in ON has resulted in an increased amount of ON measures. Nowadays, there are ten distinct ON measures [22], including the Bratman Orthorexia Test [3], the Dusseldorf Orthorexia Scale [23], the Teruel Orthorexia Scale [24] and the ORTO-15 [25]. Despite being criticized for its psychometric properties [26], low internal consistency and limited content validity [11], the ORTO-15 remains the most commonly used tool for the assessment of ON [25]. A revised and more refined format of the ORTO-15, the ORTO-R [27], was recently introduced by Rogoza and Donini aiming at a more precise measurement of ON.

Since screening for orthorexia nervosa is now part of clinical practice, the measurement instruments to be used must be clinically significant, reliable, valid and sensitive to our target population [28]. Nowadays there is no evidence about the relationship between ON (measured by ORTO-R) and DEA among general population. The main objective of the present study was to confirm the factor structure of the Arabic version of the ORTO-R using a first sample of Lebanese adults and confirm those results on another sample. The secondary objective was to assess sex differences in terms of ON and disordered eating attitudes (DEA) as well as to examine whether symptoms of ON were related to DEA in Lebanese adults.

## Methods

### Ethics approval and consent to participate

The present study has been approved by the ethics committee at the Psychiatric Hospital of the Cross. All procedures performed in our study were in accordance with the 1964 Helsinki declaration (adopted by the 18^th^ World Medical Association General Assembly, Helsinki, Finland) and its later amendments or comparable ethical standards. Written informed consent was obtained from all participants. Participants received no financial incentive.

### Participants and study design

Lebanese adults were selected to participate in this cross-sectional study (January-May 2018) using a proportionate random sample from all Lebanese governorates. The latter is divided in sub-districts, which are also divided in villages. In each selected village, the questionnaire was distributed randomly to the households, based on random sampling technique to select the included house. All members in the household, if eligible, were invited to participate in the study; those who accepted our invitation were asked to fill out the questionnaire. Persons suffering from a clinical mental impairment (as reported by a family member) affecting their ability to understand the questions were excluded from the study. The same methodology is described elsewhere in papers from the same project [19, 29–32]. The study included 783 participants (33.5% females). The mean age of the total sample was 27.78 ± 11.60 years (Min. 18 –Max. 84) and their mean BMI was 24.36 ± 5.31 kg/m^2^. Moreover, 25.3% of the total sample had inappropriate eating attitude (scored above clinical cut-off on the EAT-26) (Table 1).

**Table 1:** Characteristics of participants.

Variables		Lebanese sample (N = 783)		
		N	%	M±SD
Sex	Female	270	33.5	-
	Male	536	66.5	-
Age (years)	-	-	-	27.78 ± 11.60
BMI (kg/m^2^)	-	-	-	24.36 ± 5.31
Ranges of BMI	< 18.5	62	8.2	-
	18.5–24.99	403	53.4	-
	25–29.99	203	26.9	-
	≥ 30	87	11.5	-
EAT-26% scoring above cutoff (≥ 20)		198	25.3	

Numbers might not add to the total N because of missing values.

### Minimal sample calculation

A total of 60 participants was deemed necessary the factor analysis of the ORTO-R scale (10 observations per scale item) [33], whereas 107 participants were needed to conduct the bivariate analysis based on a correlation of 0.363 between EAT-26 and ORTO-R [34], a risk of error or 2% and a power of 95% according to the G-Power software.

### Measures

Demographic information was gathered, including participants’ self-reported sociodemographic characteristics such as age, sex and anthropometry (self-reported weight and height were used to calculate Body Mass Index). BMI was categorized according to the World Health Organization (WHO) recommendations: underweight <18.5 kg/m^2^, normal weight between 18.5 and 24.9 kg/m^2^, overweight between 25 and 29.9 kg/m^2^, and obesity ≥ 30 kg/m^2^.

#### Assessment of orthorexia nervosa: ORTO-R

The ORTO-R [27], the revised version of ORTO-15 [25], assesses ON thoughts and behaviors. Due to unstable factorial structure of the ORTO-15 across different populations, authors suggest using the ORTO-R instead. This revised scale consists of six items from ORTO-15, which were identified as the best markers of ON [27]. Those items were scored on a four-point Likert scale (never, sometimes, often and always). In present study, we used the Arabic [35] version of the ORTO-15. The ORTO-R has reversed scoring, with lower scores indicating higher tendency towards ON.

#### Assessment of disordered eating attitudes: The Eating Attitudes Test (EAT-26)

The EAT-26 [36] was designed to measure attitudes and behaviors related to disordered eating. It consists of 26 items falling into three subscales: (a) dieting which reflects restricting intake of high caloric foods and preoccupation with body image/shape; (b) bulimia and food preoccupation which describes thoughts regarding food, binging and self-induced vomiting; and (c) oral control which reflects the self-control about food intake and the perceived social pressure to gain weight. A score of 20 or above is used as a clinical cut-off and indicates inappropriate eating behaviors [36]. All subscales can be added together for an overall score or each subscale can be used separately. The EAT-26 has been validated and translated into Arabic [37]. In the current sample, Cronbach’s alpha coefficients of the EAT-26 subscales ranged from 0.726 to 0.884.

### Statistical analysis

SPSS version 25 was used to perform the statistical analysis. The total sample was randomly divided into two subsamples, using the SPSS option; the first sample, used to conduct the factor analysis, consisted of 388 participants (mean age 27.77 ± 11.35 years; 255 (65.7%) females), whereas the second sample, used to conduct the confirmatory factor analysis, consisted of 395 participants (mean age 27.79 ± 11.86 years; 261 (66.1%) females). To confirm the ORTO-R construct validity, a factor analysis (FA) using the principal analysis component was run on sample 1. To ensure the model’s adequacy, the Kaiser-Meyer-Olkin (KMO) measure of sampling adequacy and Bartlett’s test of sphericity were calculated. Factors with an Eigen value >1 were retained. Moreover, Cronbach’s alpha values were recorded for reliability analysis for the total scale and its subscales. Second, a confirmatory factor analysis (CFA) was carried out on Sample 2 using SPSS AMOS v.24. The root mean square error of approximation (RMSEA) statistic, standardized root mean square residual (SRMR), the Tucker Lewis Index (TLI) and the comparative fit index (CFI) were used to evaluate the goodness-of-fit of the model as these are the most commonly used indices [38]. Values of RMSEA of 0.06 or less indicate a good-fitting model and a value larger than 0.10 is indicative of a poor model [38], while TLI and CFI values greater than 0.90 indicate good model fit [38]. SRMR values <0.05 show good fitting models [39, 40].

The normality of distribution of the ORTO-R and EAT-26 scores was confirmed via a calculation of the skewness and kurtosis; values for asymmetry and kurtosis between -1 and +1 are considered acceptable in order to prove normal univariate distribution [41]. These conditions consolidate the assumptions of normality in samples larger than 300 [42]. When the sample size is sufficiently large (>300), the normality assumption is not needed at all as the Central Limit Theorem ensures that the distribution of disturbance term will approximate normality [43]. A two-way ANOVA was conducted to check for an association between the ORTO-R and EAT-26 total scores and gender. The Student t test was used to test for an association between the scores and dichotomous variables. Finally, the Pearson correlation test was used to check for an association between the scores and continuous variables. P<0.05 was deemed statistically significant.

## Results

### Factor analysis: ORTO-R among Lebanese sample

Since the extracted factors were found to be significantly correlated, the promax rotation technique was used. All items of the ORTO-R were extracted and yielded a two-factor solution with Eigenvalues > 1 (variance explained  =  50.07%; KMO = 0.570; Bartlett’s sphericity test *p*<0.001; α_Cronbach_ = 0.755) (Table 2).

**Table 2:** Factor analysis of the ORTO-R scale items using the varimax rotation among Lebanese sample.

Variable	Item number from ORTO-15	Factor 1	Factor 2
Does eating healthy food change your lifestyle (frequency of eating out, friends, etc.)?	11	0.748	
Do you believe that strict consuming only of healthy food may improve your appearance?	12	0.688	
Would you agree that eating healthy food increases your self-esteem?	10	0.667	
In the last three months, did thoughts of food make you feel guilt, ashamed and anxious?	3		0.779
Does thinking about food excessively worry you for more than three hours a day?	7		0.631
Are your rigid and restrictive dietary choices conditioned by your worry about your health status?	4		0.559
Percentage of variance explained	50.07	26.53	23.54
Cronbach’s alpha	0.755	0.787	0.704

### Confirmatory factor analysis: ORTO-R among Lebanese sample

A first confirmatory factor analysis was run, using the one-factor structure obtained in the original version [27]. The following results were obtained: the Maximum Likelihood Chi-Square  =  327.50 and Degrees of Freedom  =  9, which gave a χ2/df  =  36.39. The TLI value was 0.392, whereas the standardized RMR value was 0.120. The RMSEA value was 0.209 [95% CI 0.190–0.229] (pclose<0.001) and CFI value was 0.740 respectively, indicating a poor fit of the model.

A second confirmatory factor analysis was run on sample 2, using the structure obtained in sample 1 (two-factor solution). The following results were obtained: the Maximum Likelihood Chi-Square  =  26.894 and Degrees of Freedom  =  8, which gave a χ2/df  =  3.36. The TLI value was 0.914, whereas the standardized RMR value was 0.032. The RMSEA value was 0.077 [95% CI 0.046–0.111] (pclose = 0.07) and CFI value was 0.967 respectively, indicating a good fit of the model. Table 3 presents the coefficients with standard errors and p-values of the direct effects of variables on each other.

**Table 3:** Item descriptive statistics, standardized factor loadings, and explained variance of the ORTO-R.

Variable	Standardized factor loadings	Standard error	Explained variance	*p*
Factor 1				
ORTO 12	1		0.551	
ORTO 10	0.70	0.09	0.494	<0.001
ORTO 11	0.85	0.11	0.727	<0.001
Factor 2				
ORTO 4	1		0.496	
ORTO 7	0.50	0.11	0.251	<0.001
ORTO 3	0.74	0.13	0.551	<0.001
Factor 1 ⬌ Factor 2	0.40			<0.001

**Fig 1:**
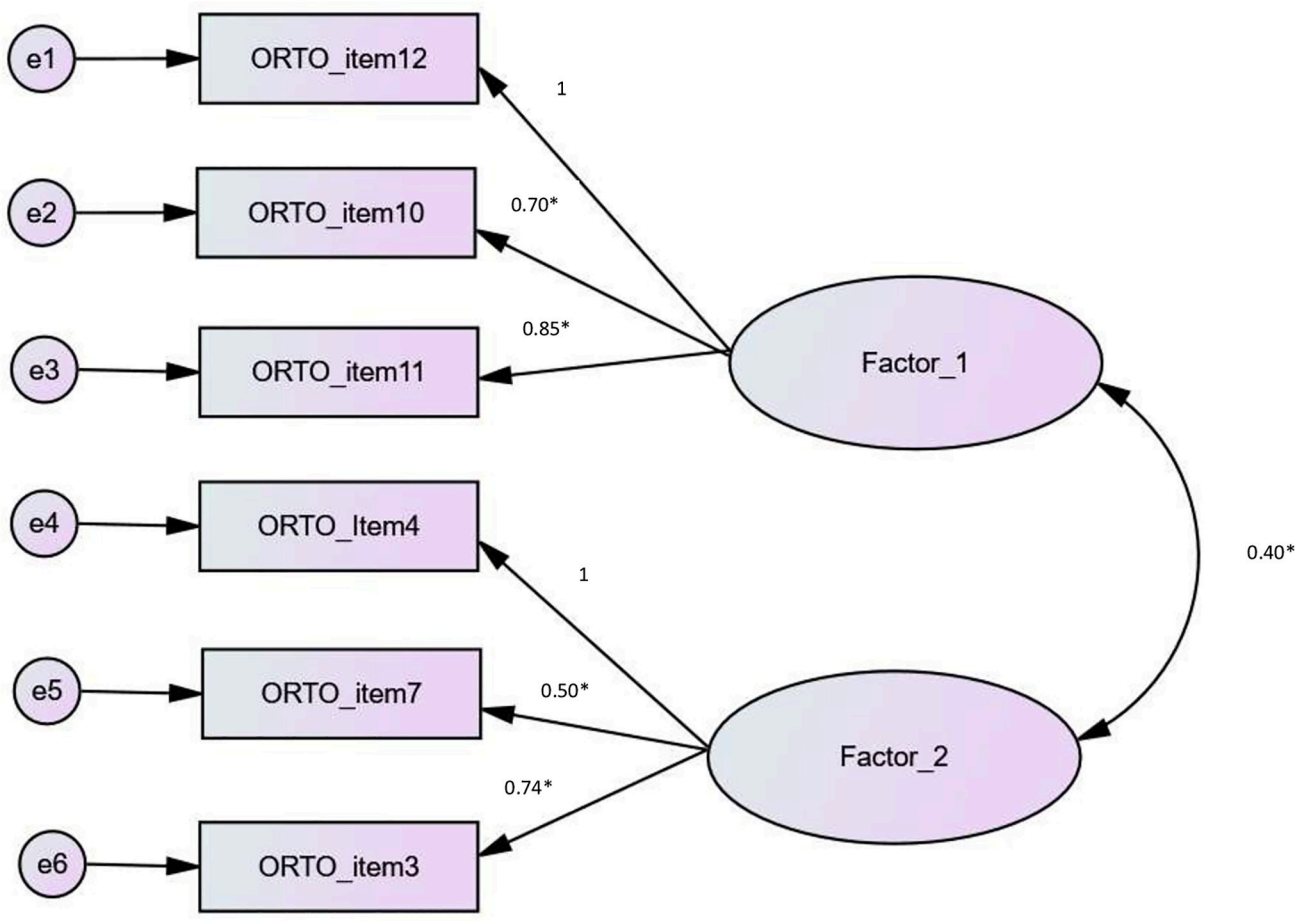
Standardized factor loadings of one factor model of the Arabic version of the ORTO-R.

The standardized factor loadings of the two-factor model of the Arabic version of the ORTO-R model are presented in Fig 1.

### Measurement invariance between males and females

To assess the degree to which the CFA results remain the same between genders, we have conducted the Multigroup Confirmatory Factor Analysis (MGCFA; estimated as CFA) between the data reported in men and in women. The results were as follows: Cmin/df = 2.404; CFI = 0.982; RMSEA = 0.043 [95% CI 0.025–0.06] (pclose = 0.741), showing that we have configural invariance. We also obtained metric invariance between genders as follows: for the unconstrained model, we obtained the following values: χ^2^ = 39.5 and df = 20, whereas for the fully constrained model, we obtained the following values: χ^2^ = 39.6 and df = 22 (p = 0.951) [44]. According to the conventional criteria of evaluation of the measurement invariance models [45], we have provided evidence of full configural and metric invariance between men and women.

### Comparison of sociodemographic characteristics (age, gender, BMI) in terms of ON and disordered eating attitudes

Female gender was significantly associated with lower ORTO-R scores compared to males (B = -0.65; p = 0.026, 95% CI -1.22- -0.08; ɳ^2^ = 0.006). However, no significant difference was found between genders in terms of EAT-26 scores (B = 0.23; p = 0.813, 95% CI -1.66–2.12; ɳ^2^ = 0.0001).

Furthermore, age was neither associated with ORTO-R (r = -0.031; p = 0.388) nor with the total EAT-26 score (r = 0.038; p = 0.297). Higher BMI was significantly but weakly associated with ORTO-R (r = 0.077; p = 0.034) and EAT-26 (r = 0.139; p<0.001) scores.

### Correlations between ORTO-R and EAT-26 scores

Higher ORTO-R score (lower/ less pathological ON tendencies and behaviors) was significantly related to higher EAT-26 total score (higher level of DEA) as well as higher dieting, bulimia and oral control scores in both females (from a weak to a moderate positive correlation) and males (a weak positive correlation) (Table 4).

**Table 4:** Correlation between ORTO-R and EAT-26 scores by sex.

Variable	Female	Male
EAT-26 dieting scale	0.501^a^	0.423^a^
EAT-26 bulimia and food	0.262^a^	0.232^a^
EAT-26 oral control	0.226^a^	0.279^a^
EAT-26 total score	0.428^a^	0.360^a^

^a^ p<0.001; in ORTO-R lower scores correspond to a more pathological behavior.

## Discussion

The present study aimed at investigating the psychometric properties of the Arabic version of the ORTO-R scale in a general population sample. The factor analysis yielded a two-factor solution in the Arabic version of the ORTO-R, which later confirmed by a CFA with a satisfactory goodness-of-fit. Reliability analysis of the ORTO-R across the whole questionnaire showed adequate internal reliability (α_Cronbach_ = 0.755). The ORTO-R was also moderately correlated to the EAT-26 total score, confirming the convergent validity of the scale. This demonstrates that the Arabic version of the ORTO-R can be considered a reliable ON screening tool for the Lebanese population. To the best of our knowledge, only one study [46] investigated the factorial structure of the ORTO-R indicating an acceptable level of model fit and a good reliability (ω  =  0.72).

The second objective was to assess sex differences in terms of ON and DEA (assessed by the EAT-26 scale). Our results demonstrated that females were more likely to have ON thoughts and behaviors than males. Recent Lebanese studies [19, 47, 48] has found that female gender is related to higher ON. Lebanese women concentrate more on eating habits, dieting and eating restraint than men [19]. Moreover, the previous study [49] found differences between Lebanese females and males in terms of their reported food intake and dietary patterns. Women adopted the vegetarian/low calorie diet (characterized mainly by consumption of plant-based food while avoiding “western” food, composite dishes, and bread) more commonly than men (70% versus 30%). The adoption of ‘healthier’ dietary patterns was related to female gender. On another hand, males were more likely to adopt a westernized diet (characterized by high consumption of white bread and western food, and a strong avoidance of plant food and composite dishes) compared to females (50% more than females). Inconsistent evidence from recent studies related to sex and ON should be taken into consideration. Some research have reported increased level of ON among women [50], others have indicated higher ON symptoms among men [51], and others have found no sex differences [52, 53].

The Lebanese culture is characterized by a collectivist culture [54]. Studies examining psychopathological traits, much of them co-morbid and/or associated with eating disorder (ED) pathology, demonstrated that individuals from collectivistic societies, compared to those from individualistic countries, reported lower self-esteem, maladaptive perfectionism (mainly high parental expectations) and depression symptoms, but also less emotional expression or suppression of emotions. Thus, it can be assumed that Lebanese present a higher risk of developing ED pathology. Based on our results we can suppose that, contrary to recent study among patients with anorexia nervosa from China, UK and Spain [55] as well as recent study among Polish and Spanish university students [56], collectivism values might increase the expression of ED pathology in Lebanon.

The present results also indicated that no significant difference was found between women and men in terms of DEA, in opposite to previous studies [57, 58] that showed that men had higher levels of DEA compared to women. These findings suggest that DEA could have a similar burden in both genders. Higher ORTO-R score (lower level of ON) was related to higher EAT-26 score (higher level of DEA) in both males and females. It may suggest that Lebanese sample may be more at increased risk for DEA than for ON as well as that DEA are not related to greater ON tendencies in this sample. The positive relationship between ON and DEA among men was found in the previous study [59]. It is worth pointing out that the literature demonstrates that past dieting experience predicts greater ON or is positively associated with greater ON tendencies [13].

The present study has some limitations. Firstly, the cross-sectional design does not allow the assessment of test-retest reliability of the Arabic version of the ORTO-R. Secondly, a cross-sectional design does not allow us to infer causality even if our findings demonstrated association between ON and DEA. The ORTO-R was measured in the current study using a four-point Likert scale (vs. a 5-point Likert scale in the original version [27]). A selection bias is possible forbidding us from generalizing our results to the general population since participants had a low mean age and genders were not represented equally. A residual confounding bias is also possible since not all factors associated with ON were taken into consideration in this paper. The sample cannot be considered representative of the general population since the majority of the participants had a university level of education and women were more represented in the sample. Finally, our data rely on self-reported measures, therefore the results may be susceptible to social desirability biases.

## Conclusions

This cross-sectional population-based study confirmed the factor structure of the Arabic version of the ORTO-R, demonstrated an association between ON and DEA and revealed more ON among females compared to males. The Arabic version of the ORTO-R seems to be an adequate instrument for ON screening among Lebanese adults, with more research needed to confirm our results. The link between ON and DEA is not yet completely understood. Future longitudinal studies are warranted for this purpose.
